# Polymodal roles of TRPC3 channel in the kidney

**DOI:** 10.1080/19336950.2020.1804153

**Published:** 2020-08-12

**Authors:** Naghmeh Hassanzadeh Khayyat, Viktor N. Tomilin, Oleg Zaika, Oleh Pochynyuk

**Affiliations:** Department of Integrative Biology and Pharmacology, The University of Texas Health Science Center at Houston, Houston, TX, USA

**Keywords:** Ca^2+^ reabsorption, osmosensitivity, urine osmolarity, Ca^2+^ signaling, renal fibrosis

## Abstract

TRPC3 is a Ca^2+^-permeable cation channel commonly activated by the G-protein coupled receptors (GPCR) and mechanical distortion of the plasma membrane. TRPC3-mediated Ca^2+^ influx has been implicated in a variety of signaling processes in both excitable and non-excitable cells. Kidneys play a commanding role in maintaining whole-body homeostasis and setting blood pressure. TRPC3 is expressed abundantly in the renal vasculature and in epithelial cells, where it is well positioned to mediate signaling and transport functions in response to GPCR-dependent endocrine stimuli. In addition, TRPC3 could be activated by mechanical forces resulting from dynamic changes in the renal tubule fluid flow and osmolarity. This review critically analyzes the available published evidence of the physiological roles of TRPC3 in different parts of the kidney and describes the pathophysiological ramifications of TRPC3 ablation. We also speculate how this evidence could be further translated into the clinic.

## Contributions of TRPC channels to renal function

**Figure 1.**
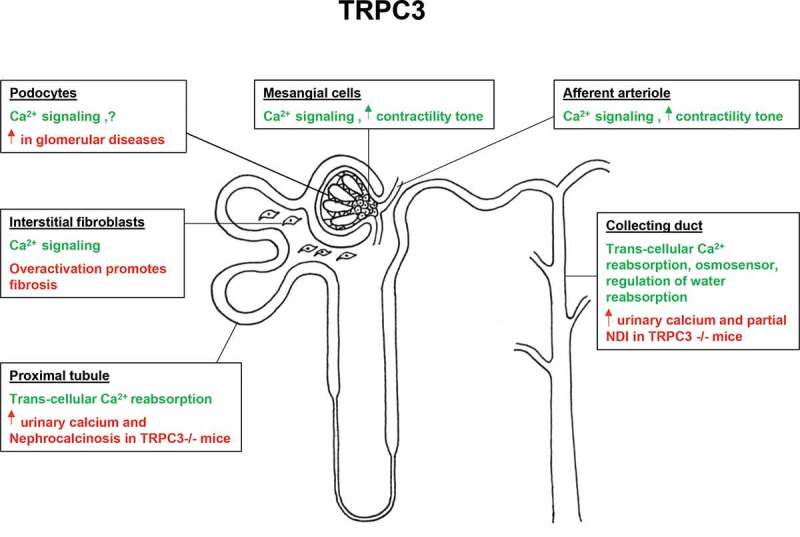
Physiology and pathology of TRPC3 in the kidney. Schematic representation of renal nephron structure with the reported sites of TRPC3 channel expression. Green color represents suggested functions/roles of the channel, while pathophysiological ramifications of TRPC3 deletion or over-activation are highlighted in red.

Located in the perirenal space of the retroperitoneum, kidneys perform numerous vital functions ranging from removal of waste products and harmful substances, maintaining the homeostasis of water and solutes, supervising acid–base balance, and control of blood pressure to the synthesis of hormones and biologically essential products. Genetic or acquired renal diseases are among the leading factors to mortality and are recognized as both major health problem and economic burden [1–3]. Moreover, chronic kidney disease reflects a serious complication of many different diseases, including diabetes, hypertension, and systemic immune disorders [4–6]. Kidneys continuously filter large volumes of plasma (typically 90 ml/min or roughly 35 times of the circulatory volume per day) through approximately 1 million of highly specialized structures called glomeruli (see Figure 1 for schematic representation of the renal nephron structure). Podocytes are terminally differentiated visceral epithelial cells that surround the glomerular capillaries with long and fine cytoplasmic extensions creating interlocking foot processes and forming slit diaphragms [7]. The slit diaphragms determine the selectivity of the glomerular filtration by limiting the passage of macromolecules based on their size and charge [7]. Loss/damage of podocytes disrupts the filtration barrier leading to proteinuria and contributes to the development and progression of chronic kidney disease [7–13]. Contractile intraglomerular mesangial cells provide structural support for the glomerular capillaries. It is viewed that the mesangial cells regulate blood flow across the glomerular capillaries thus controlling the filtration rate [14,15]. Blood is supplied to the glomeruli via high resistance afferent arterioles and the filtered fraction enters into the renal tubules, which possess highly spatial organized systems to perform transport of water and solutes until final urine is formed and ultimately excreted. Renal tubule starts with the proximal tubule (PT) segment, which reabsorbs up to 70% of the filtered load depending on the volume status, thereby setting up the pace for the consecutive parts [16]. However, PT reabsorption is auto-regulated by the tubuloglomerular feedback mechanism [16]. Following PT is the Loop of Henle which is responsible for the generation of cortical-medullary osmotic gradient to execute controlled water reabsorption by the collecting duct (CD) in order to determine the urinary volume and osmolarity [17,18]. The late tubular segments comprised of the distal convoluted tubule (DCT), connecting tubule (CNT), and the CD have much lower transport capacity (approximately 10% of the filtered load altogether), but they play major roles in maintaining water and electrolyte balance in response to variations in dietary intake [17,19–21]. All aforementioned structural components of the kidney exhibit highly sophisticated regulatory signaling machinery, membrane receptors, and mechano-responsive elements to integrate multiple endocrine and physical inputs during perpetually changing environmental and energy demands.

Transient receptor potential (TRP) channels are famous for their remarkable ability to get activated by various extracellular (mechanical stimuli, ligand operated G-protein coupled receptors (GPCR), cold and hot temperatures) and intracellular stimuli (store depletion, second messengers) [22]. TRP channel superfamily consists of TRPC (canonical, initially classical), TRPV (vanilloid), TRPM (melastatin), TRPP (polycystin), TRPA (ankyrin), TRPML (mucolipin), and TRPN (no mechanoreceptor potential C, only found in invertebrates and zebrafish) subfamilies [22,23]. While TRP channels are usually permeable to both monovalent (Na^+^, K^+^, Cs^+^) and divalent (Ca^2+^, Mg^2+^) cations, it is thought that TRP-dependent Ca^2+^ influx and subsequent [Ca^2+^]_i_ elevations are responsible for the majority of TRP channel physiological roles [23]. All TRP channels are tetramers assembled around the central pore with each subunit containing six transmembrane domains and intracellular amino- and carboxyl- termini [22,23].

TRPC channel family consists of seven members (TRPC1-7), with *TRPC2* being a pseudogene in humans, while forming a functional channel in rodents [24,25]. TRPC channels share the highest homology with the original TRP proteins initially discovered in invertebrates [26,27]. TRPC channels could be further subdivided into TRPC1, TRPC3/6/7, and TRPC4/5 groups based on the sequence homology and the ability to form functional heteromers [28]. TRPC channel activation is commonly linked to the receptor-operated calcium entry (ROCE) and in some instances to the store-operated calcium entry (SOCE) [29]. Activation of GPCR (most commonly coupled to G_q/11_) and receptor tyrosine kinase (RTK) leads to stimulation of phospholipase C (PLC) which in turn breaks the membrane-associated phosphatidylinositol 4,5-bisphosphate (PI(4,5)P_2_) and releases diacylglycerol (DAG) and inositol 1,4,5-trisphosphate (IP_3_). DAG could directly stimulate TRPC channels or affect their activity via protein kinase C (PKC) [30]. IP_3_ causes Ca^2+^ release from the endoplasmic reticulum (ER) stores. ER store depletion triggers TRPC-dependent Ca^2+^ influx via SOCE mechanism. However, SOCE is much more commonly associated with stimulation of the highly Ca^2+^ selective Orai1-3 channels rather than nonselective TRPC channels [31].

Expression of several TRPC channels, namely TRPC1, TRPC3, TRPC5, and TRPC6, has been reported in the renal tissue [32,33]. Specifically, TRPC1 and TRPC3 could be found in the PT [32,34]; TRPC3 and TRPC6 are present in the renal vasculature, glomerulus, and the CD [32,35]; TRPC5 expression seems to be restricted to podocytes in the glomerulus [33]. Such a broad distribution suggests that TRPC channels are likely playing important roles in ROCE and SOCE signaling in response to systemic hormonal, such as Ang II, or local, for example, ATP and adenosine, signals. Indeed, gain-of-function mutations of TRPC6 have been demonstrated to underlie the Focal Segmental Glomerulosclerosis (FSGS) in humans [10,36]. FSGS is characterized by proteinuria, hypoalbuminemia, hypertension, and decreased renal function progressing to the end-stage renal disease in approximately 40% of patients [37]. It is viewed that sustained hyper-activation of TRPC6 channels results in [Ca^2+^]_i_ overload leading to effacement and ultimately podocyte loss. The decrease in podocyte population is further exacerbated due to their limited capacity for regeneration. It was also noted that the magnitude of TRPC6 potentiation correlates with the severity of disease progression [38,39]. In addition, it was demonstrated that over-activation of TRPC6 by Ang II and purinergic signaling causes podocyte hypertrophy and foot process effacement thereby contributing to the development of diabetic kidney disease [40,41]. Conversely, TRPC6 deficient rodent models demonstrate marked protection against glomerular damage [42–44]. It is argued that selective TRPC6 inhibition might be of clinical relevance to treat FSGS [45]. At the same time, the current research focus on TRPC6, while essential and clinically relevant, outshines our appreciation for the roles of other TRPC channels in the kidney. This is particularly the case for TRPC3, being a TRPC6’s “half-brother” both numerically and with regard to its significance. However, TRPC3 and TRPC6 have very similar modes of activation and expression patterns in renal tissue. The current review attempts to tilt the scale by describing recent advances in understanding different roles of TRPC3 and highlight its significance for renal function.

## Molecular aspects of TRPC3 function

TRPC3 is a nonselective cation channel with permeability to Ca^2+^ versus Na^+^ being estimated as 1.6 [46]. Similar to other members of the canonical subfamily, TRPC3 activation is linked to stimulation of GPCR followed by stimulation of the downstream phospholipase C (PLC)-dependent cascade [47–50]. Mechanistically, PLC-induced PI(4,5)P_2_ hydrolysis to yield diacylglycerol (DAG) has been instrumental for the activation of TRPC3 in many cell types [51–53]. Cryo-EM 3-D structure of TRPC3 provided many important insights into its molecular organization and revealed several anatomical features, which potentially underlie its unique biophysical profile. TRPC3 has two lipid-binding pockets, which are likely the sites of direct DAG binding: in the large elbow-like domain just prior to the first transmembrane segment and in the lateral fenestration of the pore domain, wedged between the P-loop and 6^th^ transmembrane domain of the adjacent subunit [54]. In addition, the unusually long third transmembrane segment of the channel protrudes into the extracellular space to form a pocket-like domain [54]. It is reasonable to assume that this structural feature is well suited for the regulation of TRPC3 by external cues and mechanical forces. Indeed, mechanical activation of TRPC3 has been well documented in the literature (reviewed in [41,55]). Interestingly, this extracellular pocket domain is absent in DAG-insensitive TRPC1, TRPC4, and TRPC5, but is also present in DAG-sensitive TRPC6. Such correlation points to a functional convergence between DAG and mechanosensitive modes of TRPC3 activation. In addition, TRPC3 is also a subject of regulation by different posttranslational modifications. Src tyrosine kinase-dependent phosphorylation of Y226 on N-terminus of TRPC3 leads to increased channel activity [56]; whereas protein kinase C (PKC)-induced phosphorylation at S712 on C-terminus is inhibitory [57].

## TRPC3 roles in renal vasculature and glomerulus

Accrued experimental evidence suggest a broad TRPC3 distribution in the kidney. Thus, TRPC3 expression has been reported in both renal epithelia and vasculature. TRPC3 is the most abundant TRPC channel in the pre-glomerular resistance vessels aka afferent arterioles, where it contributes to the Ca^2+^ signaling and receptor-operated Ca^2+^ entry in the vascular smooth muscle cells [58]. The magnitude of the adenosine receptor type 1 (A1R)-stimulated [Ca^2+^]_i_ elevations positively correlates with TRPC3 expression in afferent arterioles of postnatal and 20 days old piglets [59]. Moreover, activation of the calcium-sensing receptor (CaSR) by high extracellular Ca^2+^ augmented proliferation of glomerular contractile mesangial cells via stimulation of TRPC3-mediated Ca^2+^ influx [60]. Overall, TRPC3 is likely a factor for setting up the glomerular filtration rate (GFR) and maintaining the tubuloglomerular feedback (TGF), but direct evidence is currently lacking.

Notable TRPC3 expression has been demonstrated in the glomerular podocytes [32,61]. However, very little is known about the physiological role of the channel in these cells. In contrast to TRPC6, there are no reported TRPC3 mutations associated with FSGS or other glomerular disease to our knowledge. However, augmented TRPC3 expression has been found during different pathophysiological states, such as Angiotensin II–induced hypertension and glomerulonephritis [62,63]. Furthermore, there is a notable upregulation of TRPC3 levels in podocytes from TRPC6 deficient rodent models [42,62]. This indicates that at least some TRPC3 and TRPC6 functions may overlap in the glomerulus. However, the diligent examination of the interplay and supremacy of TRPC3- versus TRPC6-dependent Ca^2+^ influxes for podocyte function and pathophysiology is still a task to complete.

## TRPC3 significance in the proximal tubule and renal interstitium

Along the renal tubule, TRPC3 expression has been found in the proximal tubule (PT) and the collecting duct (CD) system [32,34]. In the PT, TRPC3 is localized to the brush border area of the epithelial cells. Physical and functional coupling of TRPC3 and the calcium-sensing receptor (CaSR) has been shown to participate in trans-cellular Ca^2+^ reabsorption at this site [34]. It was further proposed that activation of CaSR and subsequent TRPC3-dependent Ca^2+^ influx affects luminal Ca^2+^ levels to regulate urinary Ca^2+^ excretion. TRPC3 knockout mice exhibited hypercalciuria and microcalcifications arguing for a protective role of TRPC3 in preventing nephrocalcinosis (renal stone formation) [34]. However, careful investigation of the renal Ca^2+^ clearance in animal models with proximal tubule-specific TRPC3 deletion is necessary to define the significance of the proximal tubule transcellular versus paracellular Ca^2+^ reabsorption as well as to sort out the contribution of the TRPC3-mediated Ca^2+^ transport in the distal nephron.

TRPC3 expression has also been identified in the interstitial fibroblasts of the renal cortex. Unilateral ureteral obstruction led to increased TRPC3 expression in fibroblasts in the manipulated but not in the control kidney [64]. Augmented TRPC3-dependent influx was further linked to the increased phosphorylation of the extracellular signal-regulated kinase 1/2 (ERK1/2) to stimulate fibroblast proliferation leading to renal fibrosis. TRPC3 blockade in rats and TRPC3 knockout in mice inhibited ERK1/2 phosphorylation and fibroblast activation, as well as myofibroblast differentiation and extracellular matrix remodeling in obstructed kidneys, thus ameliorating tubule-interstitial damage and renal fibrosis [64].

## TRPC3 regulates Ca^2+^ reabsorption in the collecting duct

In the distal nephron, TRPC3 expression was found in the apical and intracellular compartments of the AQP2-positive cells in both cortical and medullary segments [65]. However, there was no co-localization of TRPC3 with the sodium-calcium exchanger (NCX), which is a marker of the distal convoluted tubule (DCT) and the connecting tubule (CNT) also expressing AQP2. Altogether, this suggests that TRPC3 is restricted to the principal cells of the CD. At the same time, TRPC3 was not co-localized with AQP1, the marker of PT cells [65]. While there is no agreement about the profile of TRPC3 expression in the renal nephron reported by different groups [34,65], it is reasonable to assume that TRPC3 is functional in both PT and CD. The available published evidence demonstrated prominent TRPC3-dependent Ca^2+^ influxes in native freshly isolated cells from both segments, which were dramatically decreased upon TRPC3 genetic deletion [34,35].

TRPC3 expression and activity in the CD principal cells are also subjects of regulation by GPCR signaling. Thus, elevated circulating levels of the arginine vasopressin (AVP) cause translocation of TRPC3 to the apical plasma membrane via stimulation of the vasopressin V2 receptors (V2R) [65]. The downstream cascade involves activation of the adenylyl cyclase (AC)/cAMP/protein kinase A (PKA) signaling in the AVP-induced TRPC3 trafficking [66]. Moreover, increased AVP levels after 24 h water deprivation augmented TRPC3-dependent Ca^2+^ influx in freshly isolated split-opened CDs in WT but not in TRPC3 -/- mice [35]. Apical application of adenosine triphosphate (ATP) also stimulated TRPC3 activity in inner medullary CD cells [67] and to a lesser extent in cortical CDs [68]. ATP-induced [Ca^2+^]_i_ elevations were dependent upon the stimulation of purinergic P2Y2 receptors and downstream PLC cascade [68]. It was further shown that activation of TRPC3 activity by the apical ATP led to the net apical-to-basolateral Ca^2+^ flux in inner medullary CD cells [67], indicating that TRPC3 is instrumental in mediating transcellular Ca^2+^ reabsorption at this site. Indeed, the CD possesses a notable Ca^2+^ reabsorption [69] despite being often unrightfully discarded as a contributor to the renal Ca^2+^ handling. In addition, anti-calciuretic effects of AVP have been described in the literature [70]. It is reasonable to assume that AVP stimulates Ca^2+^ reabsorption in the CD by promoting TRPC3 translocation to the apical membrane [65]. This, in turn, would prevent accumulation and precipitation of Ca^2+^ in the CD during avid water reabsorption in the state of antidiuresis. Interestingly, hypercalcemia in the Williams-Beuren syndrome (WBS) has been associated with augmented TRPC3 expression in both collecting duct and intestinal epithelium due to mutation in the transcription factor TFII-I [71]. Furthermore, the potential role of TRPC3 in mediating systemic Ca^2+^ balance is supported by the higher urinary Ca^2+^ excretion in TRPC3 -/- mice [34], likely due to reduced Ca^2+^ reabsorption in both PT and CD [34,67]. It is somewhat unexpected that parathyroid hormone (PTH) levels did not change upon TRPC3 deletion and serum Ca^2+^ levels did not decrease in the knockouts [34]. This could indicate disturbed Ca^2+^ resorption-deposition in the skeleton. Indeed, TRPC3 is expressed in osteoblastic cells, where it participates in the store-operated nonselective cation entry under control of 1α, 25(OH)_2_D_3_ [72]. This further supports a multifactorial role of TRPC3 in shaping systemic Ca^2+^ homeostasis, as the channel is functionally expressed in the kidney, intestine, and bones – the major tissues responsible for Ca^2+^ balance.

## TRPC3 serves as osmosensor in the CD

It is generally viewed that the CD is the principal site for controlled renal water transport via AVP-driven trafficking of AQP2 to the apical plasma membrane and stimulation of AQP2 expression [73,74]. Disruption of this mechanism results in a devastating pathology of Nephrogenic Diabetes Insipidus (NDI), associated with impaired urinary concentrating ability, excretion of large volumes of hypotonic urine, plasma hypertonicity, and secondary polydipsia in humans and rodents [75,76]. It has long been recognized that in addition to cAMP, AVP increases [Ca^2+^]_i_ in the CD cells by activation of the ryanodine – but not IP_3_-dependent stores followed by store-operated Ca^2+^ entry (SOCE) [77–79]. Our group has recently reported that disruption of the SOCE due to truncation mutation in the endoplasmic reticulum Ca^2+^ sensor, STIM1 compromises AVP-induced [Ca^2+^]_i_ signaling [79]. This leads to decreased AQP2 abundance and its intracellular retention suggesting impaired sensitivity of the CD to AVP, manifesting as a partial NDI [79]. However, reports from cultured IMCD cells and freshly isolated split-opened CDs did not support a contribution of TRPC3 in SOCE, which was mediated by the highly Ca^2+^ selective Orai1 channel [67,79].

It is important to emphasize that the AQP2-dependent water reabsorption in the CD occurs only in the presence of a favorable osmotic gradient with luminal fluid being hypotonic when compared to the cytoplasm. At the same time, this osmotic difference generates a mechanical stress on the apical membrane. Mechanosensitivity of the tubular cells has long been recognized to regulate water and electrolyte transport, the rate of proliferation, etc. [80–82]. The CD cells constantly experience substantial variations in tubular flow rate and changes in osmolarity as a result of variations in dietary electrolyte and water intake [82,83]. It is viewed that the magnitude of these mechanical forces is sensed in the form of elevated [Ca^2+^]_i_ to initiate proper cellular response(s) [84]. Using both pharmacological inhibition and genetic knockout, our group has demonstrated a central role of TRPC3 in mediating hypotonicity-driven Ca^2+^ influx in native CD cells [35]. It appears that TRPC3-dependent Ca^2+^ influx likely leads to the secondary Ca^2+^ release from the intracellular stores to further augment the osmosensitive signal. However, removal of the extracellular Ca^2+^ or TRPC3 deletion abolishes hypotonic induced [Ca^2+^]_i_ elevations in native CD cells regardless of the status of intracellular Ca^2+^ stores [35]. This provided direct evidence that activation of TRPC3 mediates the initial step in driving osmosensitive [Ca^2+^]_i_ elevations in CD cells. However, it is not known whether TRPC3 could be directly activated by a mechanical stretch of the plasma membrane due to unique structural features of the channel, such as extra-long S3 transmembrane segment [54]. Alternatively, TRPC3 could be activated in response to a plasma membrane-associated signaling cascade, such as PLC, commonly reported in the literature [30].

It is remarkable that mechanosensitive activation of TRPC3 is restricted to the hypotonicity-induced cell swelling/stretch in the CD cells. Our group as well as others provided compelling evidence that the activity of Ca^2+^-permeable TRPV4 channel is central for [Ca^2+^]_i_ elevations in the CNT/CD cells in response to increased tubular flow [85–88]. Interestingly, a recent study demonstrated a distinct effect of the fluid shear stress (corresponding to high tubular flow) and circumferential stretch (reflecting osmosensitive cell swelling) on stimulation of ERK and p38 cascades and PGE2 release in CD cells [89]. Thus, it is reasonable to propose that CD cells are capable of distinguishing between different types of mechanical stress by activating either TRPC3 or TRPV4 to drive a condition-specific downstream cascade. Consistently, stimulation of tubular flow in the CD by feeding mice with a high K^+^ diet drastically increased TRPV4 expression and activity, whereas this treatment had no effect on TRPC3 expression [86]. Conversely, TRPV4 deletion did not affect [Ca^2+^]_i_ responses to hypotonicity in freshly isolated split-opened CDs [35]. Moreover, TRPV4 -/- mice have normal plasma tonicity and urinary volume/osmolarity at the baseline and after water deprivation [90,91], suggesting that TRPV4 is not involved in osmosensitivity in the CD.

## Contribution of TRPC3 to water transport in the collecting duct and urinary concentrating ability

**Figure 2.**
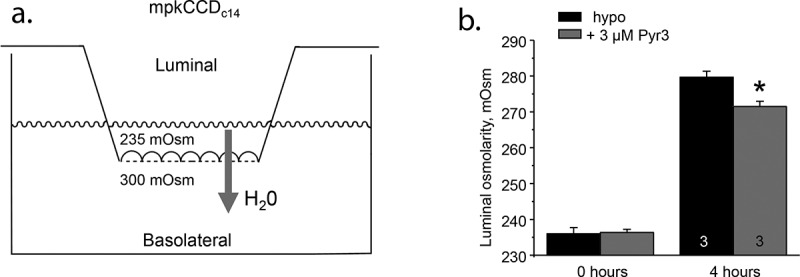
Inhibition of TRPC3 diminishes water transport in mpkCCD_c14_ cells. (a) Schematic representation of the experimental design for measurement of the apical-to-basolateral water flux in high resistance polarized monolayers of mpkCCD_c14_ cells grown on semipermeable supports. The medium from the apical compartment (luminal side) was replaced with hypotonic medium (235 mOsm) to create an osmotic gradient. The direction of water movement is indicated with arrow. (b) Summary graph comparing osmolarity on the luminal (apical) side immediately after medium replacement and followed 4 hours of the treatment in the absence (hypo) and the presence of TRPC3 inhibitor, Pyr3 (3 µM). * – significant decrease versus respective hypo value.

AVP-stimulated TRPC3 trafficking along with channel involvement in osmosensitivity argues for its role in modulation of water reabsorption by the CD cells. The initial support of this idea came from our studies, in which we grew mpkCCD_c14_ cells on semipermeable supports until they formed polarized tight high resistance monolayers. Apical-to-basolateral water movement was induced by the introduction of the hypotonic apical media (~80% of control osmolarity) and was quantified as restoration of the luminal osmolarity over time (Figure 2(a)). TRPC3 inhibition with Pyr3 (3 µM) on the apical side resulted in a significantly lower luminal osmolarity after 4 hours compared to vehicle treatment, which is indicative of the reduced water transport (Figure 2(b)). To verify the significance of this effect, our group further employed a concomitant assessment of the TRPC3-dependent [Ca^2+^]_i_ signaling and cell volume changes in freshly isolated split-opened CDs [35]. It has been shown that the rate of hypotonicity-induced cell swelling is proportional to water permeability [92] and thus could be used as a reliable readout of AQP2 expression on the plasma membrane. TRPC3 deletion abolished [Ca^2+^]_i_ responses to hypotonicity and markedly slowed osmosensitive volume changes in CD cells [35]. Furthermore, 24 h water deprivation notably increased osmosensitive [Ca^2+^]_i_ elevation and further accelerated rate of cell swelling [35] consistent with both AQP2 and TRPC3 trafficking to the plasma membrane [65]. In contrast, there was no [Ca^2+^]_i_ response to hypotonicity and only a moderate increase in cell swelling rate in water-deprived TRPC3 -/- mice [35].

**Figure 3.**
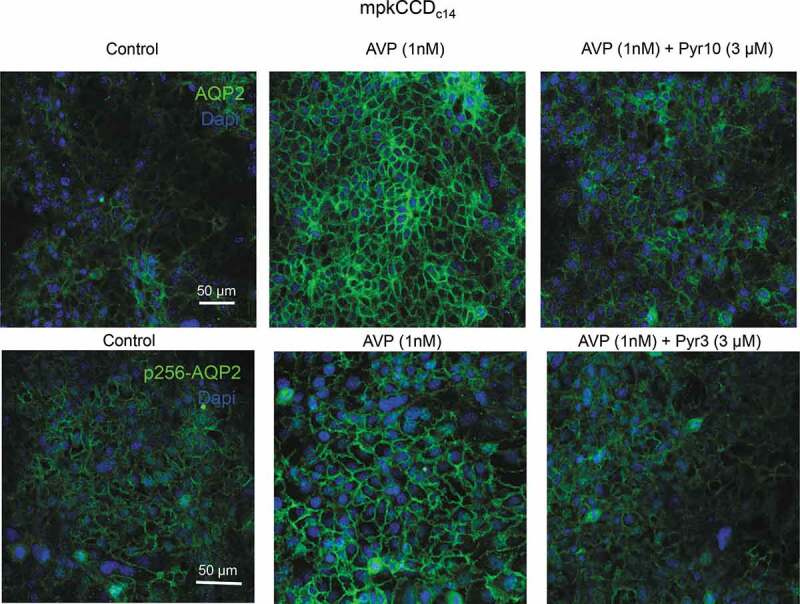
TRPC3 inhibition compromises AVP-induced AQP2 trafficking to the plasma membrane in mpkCCD_c14_ cells. Representative micrographs of confocal planes monitoring AQP2 (top panel) and phosphorylated p256-AQP2 (bottom panel) distribution in confluent monolayers of mpkCCD_c14_ cells in control, after treatment with AVP (1 nM) for 24 hours, and after AVP in the presence of TRPC3 inhibitors, Pyr10 (3 µM) or Pyr3 (3 µM). AQP2 and p256-AQP2 localizations are shown in pseudocolor green. Nuclear DAPI staining is shown in pseudocolor blue.

It also appears that TRPC3-dependent Ca^2+^ influx is necessary for AQP2 trafficking to the apical membrane in CD cells. As exemplified in Figure 3, treatment with AVP caused a marked redistribution of both anti-AQP2 and anti-p256 AQP2 (AVP-induced phosphorylated form) fluorescent signals to the plasma membrane in cultured mpkCCD_c14_ cells. Concomitant application of AVP with either of TRPC3 inhibitors (Pyr10 or Pyr3) markedly impaired AQP2 translocation (Figure 3 right panel). Consistently, AQP2 exhibited preferentially cytosolic localization and diminished translocation to the apical membrane in response to water deprivation in CDs from both cortical and medullary sections of TRPC3 -/- mice [35]. While WT and TRPC3 -/- mice have comparable urinary output and osmolarity, the knockout exhibited impaired adaptation to water deprivation. This manifested as increased urinary volume, diminished urinary concentrating ability, and serum hypertonicity despite significantly higher AVP levels in TRPC3 -/- versus WT mice [35]. Altogether, this strongly suggests that TRPC3 serves as a convergent point between osmosensitive [Ca^2+^]_i_ signaling and AQP2-water transport in the CD to regulate renal water handling and systemic water balance.

## Summary and clinical perspective

TRPC3 is a Ca^2+^-permeable channel, which is known to be activated in response to GPCR signaling as well as mechanical distortions of the plasma membrane. It is broadly expressed in the kidney where it is well suited to participate in a variety of signaling and transport processes (summarized in Figure 1). While no known TRPC3 mutations have been associated with renal diseases so far, the accumulating experimental evidence in rodent models supports the physiological relevance of TRPC3 for kidney function and provides the rationale for using TRPC3-based pharmacology in the certain pathophysiological settings. Thus, TRPC3-mediated Ca^2+^ influx contributes to maintaining the contractility tone of afferent arterioles and glomerular mesangial cells, which could be instrumental in regulating the rate of filtration. Understanding why the upregulation of TRPC3 expression is not as detrimental for podocyte function as increased TRPC6 might be of use in designing new strategies to fight diabetic nephropathy and glomerulonephritis in clinic. Inhibition of TRPC3-dependent Ca^2+^ influx in the interstitial fibroblasts has been shown to reduce fibrosis and thus could be beneficial during ischemia-reperfusion kidney injury and acute kidney failure. Strong evidence also implicates TRPC3 in mediating trans-cellular Ca^2+^ reabsorption in the proximal tubule and collecting duct. Stimulation of this mechanism might reduce the formation of renal stones. Finally, TRPC3 functions as an osmosensor in the collecting duct to regulate AQP2-dependent water reabsorption and subsequently urine volume/osmolarity. It is conceivable to speculate that TRPC3 stimulation might facilitate AQP2 trafficking to ameliorate the copious urine production during congenital or lithium-induced diabetes insipidus.
